# Argon Plasma Coagulation as Rescue Endoscopic Hemostasis for Acute Variceal Bleeding in Cirrhosis: A Retrospective Cohort Comparison with Band Ligation

**DOI:** 10.3390/medicina62030547

**Published:** 2026-03-16

**Authors:** Ilie Marius Ciorba, Nicoleta Crăciun Ciorba, Simona Maria Bățagă

**Affiliations:** 1 Department of Internal Medicine, “George Emil Palade” University of Medicine, Pharmacy, Science and Technology of Târgu Mureș, 540142 Târgu Mureș, Romania; ilie-marius.ciorba@umfst.ro (I.M.C.); simona.bataga@umfst.ro (S.M.B.); 2 Institution Organizing University Doctoral Studies (IOSUD), “George Emil Palade” University of Medicine, Pharmacy, Science and Technology of Târgu Mureș, 540142 Târgu Mureș, Romania; 3 Department of Gastroenterology, Mureș County Emergency Clinical Hospital, 540136 Târgu Mureș, Romania; 4 Department of Internal Medicine, Mureș County Emergency Clinical Hospital, 540136 Târgu Mureș, Romania

**Keywords:** acute variceal bleeding, cirrhosis, argon plasma coagulation, endoscopic band ligation, rebleeding, mortality, risk stratification

## Abstract

*Background and Objectives*: Acute variceal bleeding (AVB) in cirrhotic patients remains associated with considerable early rebleeding and mortality despite guideline-based therapy. Endoscopic band ligation (EBL) is recommended as first-line therapy for esophageal variceal bleeding, while alternative endoscopic hemostasis strategies may be required when EBL is technically difficult or judged unsafe. *Materials and Methods*: We conducted a single, tertiary referral center retrospective cohort study of adults with cirrhosis and AVB undergoing emergency endoscopy. Hemostasis modality at index endoscopy was EBL or argon plasma coagulation (APC), used selectively at the endoscopist’s discretion when bleeding was sourced to gastric varices or when EBL was technically difficult or unsafe. The primary endpoint was 5-day rebleeding, with key secondary endpoints set as 6-week mortality and in-hospital mortality. ICU admission and time to endoscopy were evaluated as process and outcome metrics. Multivariable models were used, adjusted for liver severity (MELD-Na, ALBI, PALBI) and bleeding and mortality scores (AIMS65, Rockall, Glasgow Blatchford). *Results*: Among 181 eligible AVB cases (APC *n* = 29, EBL *n* = 152), 5-day rebleeding was higher with APC (31%) than EBL (13.8%). In-hospital mortality (APC 20.7% vs. EBL 23.0%) and 6-week mortality (APC 31.0% vs. EBL 35.5%) were similar. In adjusted models (age, MELD-Na, time to endoscopy), APC was associated with increased odds of 5-day rebleeding (aOR 2.73, 95% CI 1.06–7.03), but not with in-hospital (aOR 0.51) or 6-week mortality (aOR 0.45). Time to endoscopy was not independently associated with mortality in adjusted models. Discrimination for in-hospital mortality was highest for MELD-Na (AUC 0.898) and ALBI (AUC 0.859). *Conclusions*: In this observational AVB cohort, APC, used as a rescue or alternative strategy, showed similar short-term mortality compared with EBL after adjustment for liver severity and was associated with higher 5-day rebleeding. APC may be a pragmatic option when EBL is not feasible or is judged unsafe. However, prospective evaluation and careful selection are warranted.

## 1. Introduction

Acute variceal bleeding (AVB) is a life-threatening complication of portal hypertension in cirrhosis. Contemporary management guidelines associate vasoactive agents, antibiotics, early endoscopy, and endoscopic therapy with endoscopic band ligation (EBL) recommended for acute esophageal variceal bleeding [1,2,3]. Gastric variceal bleeding and complex bleeding scenarios (massive hemorrhage with poor visualization or post-banding ulcer bleeding) may limit the feasibility or safety of EBL, prompting the use of alternative endoscopic hemostasis strategies as rescue therapy. Argon plasma coagulation (APC) is a non-contact thermal electrosurgical technique indicated for superficial mucosal bleeding (vascular ectasias, flat oozing lesions, or portal hypertensive gastropathy) [4], and it has been studied as an option in variceal eradication protocols and in the management of post-banding bleeding. However, in some centers, APC is occasionally used in clinical practice as an initial hemostatic strategy when banding is technically challenging, bleeding is diffuse, or concomitant peptic lesions are discovered. Comparative data on efficacy between APC and EBL for short-term mortality in AVB are limited [5,6,7,8]. In our tertiary referral center, APC was used as the primary recorded endoscopic hemostasis modality in 29/181 (16.0%) AVB episodes during 2024–2025, reflecting selective rescue use when EBL was not feasible or was judged unsafe. Current guidance emphasizes that definitive therapy should be tailored to the variceal type. EBL remains first-line for esophageal varices, while bleeding gastric varices are generally managed with tissue adhesive injection (e.g., N-butyl-2-cyanoacrylate) and, in experienced centers, EUS-guided therapies (coil ± cyanoacrylate) to improve obliteration and reduce rebleeding, with consideration of early or rescue TIPS or transvenous obliteration techniques when appropriate [2,9,10].

We aimed to compare early rebleeding and short-term mortality between APC (rescue/alternative strategy) and EBL (standard-of-care) in adults with cirrhosis and endoscopically confirmed AVB, accounting for time to endoscopy and established liver disease severity and bleeding risk scores. We also assessed discrimination of commonly used scores for in-hospital mortality and estimated adjusted mortality risk differences to explore whether an APC strategy could represent a valid endoscopic option in selected AVB cases.

## 2. Materials and Methods

We performed a single-center, retrospective cohort study including consecutive adult patients with established cirrhosis who underwent emergency upper gastrointestinal endoscopy for suspected acute upper gastrointestinal bleeding. The study period was 1 January 2024 to 31 December 2025, in a tertiary referral hospital with 24/7 endoscopy capability. Eligible cases were those with endoscopically confirmed acute variceal bleeding (AVB) at the index emergency endoscopy and treated endoscopically with either endoscopic band ligation (EBL, standard-of-care) or argon plasma coagulation (APC, rescue or alternative strategy). The study is reported in accordance with the STROBE statement [11].

We included patients aged 18 years or older with established cirrhosis (clinical, radiologic, laboratory, and/or prior specialist diagnosis) and endoscopically confirmed AVB at the index emergency endoscopy. Endoscopic confirmation required active bleeding from esophageal or gastric varices or stigmata of recent variceal hemorrhage with intragastric blood and no alternative lesion explaining the bleeding. Patients were included if endoscopic hemostasis at index endoscopy was performed using EBL or APC as the primary recorded hemostatic technique.

We excluded episodes in which variceal bleeding was not confirmed endoscopically, endoscopic therapy was not performed, or hemostasis was achieved solely using other modalities (balloon tamponade or self-expanding metal stent placement without EBL or APC). Episodes with insufficient documentation to classify exposure or evaluate outcomes were also excluded.

To minimize within-patient correlation, we analyzed one episode per patient and retained the first eligible admission during the study period.

Endoscopic hemostasis modality was classified as EBL or APC based on the index endoscopy report. EBL was the institutional standard first-line endoscopic therapy for esophageal variceal hemorrhage. APC was used selectively at the endoscopist’s discretion when bleeding was sourced to gastric varices, where ligation was not considered optimal, or to esophageal varices when band ligation was technically difficult or judged unsafe, including poor visualization due to significant bleeding or post-banding ulcer bleeding. When more than one endoscopic technique was used during the index procedure, cases were classified according to the primary hemostatic modality recorded in the endoscopy report. Conversion to a rescue modality and the sequence of therapies were documented when described.

Endoscopy was performed by using an EVIS EXERA II video endoscopy system (CV-180 video processor and CLV-180 xenon light source) with a standard forward-viewing gastroscope (GIF-H180; Olympus Medical Systems, Tokyo, Japan). EBL was performed using a multi-band, single use ligation device, EzyShoot® multi-band ligator (6-shot, G-Flex Europe SRL, Nivelles, Belgium). The actively bleeding site was targeted first, followed by additional bands placed on high-risk lesions as required until hemostasis was achieved or further banding was judged unsafe.

APC was delivered through a 2.3 mm catheter using forced mode at 25–40 W with argon flow of 0.4 L/min, using an ARC 200 HF generator with ARC PLUS argon assistance unit (BOWA-electronic GmbH & Co. KG, Gomaringen, Germany). Applications were delivered without mucosal contact, approximately 2 s per application, with up to five applications per bleeding point. Power and exposure were minimized to the lowest effective settings, and cumulative application time was limited (maximum 10 s per site) to reduce deep tissue injury.

Patients received standard medical management for AVB according to local protocols aligned with consensus guidance, including vasoactive therapy and prophylactic antibiotics, transfusion, and hemodynamic support as clinically indicated. Vasoactive therapy consisted of terlipressin (Glypressin^®^, terlipressin acetate solution for injection 0.12 mg/mL, equivalent to 1 mg/8.5 mL ampoule, Ferring Pharmaceuticals Ltd., West Drayton, UK) administered at the discretion of the treating team [12]. Prophylactic antibiotic therapy was administered to all included patients, as per guidelines for bleeding in cirrhotic patients. A restrictive transfusion strategy was used with packed red blood cells administered for hemoglobin below 7 g/dL, unless otherwise clinically indicated (patient instability, poor clinical tolerance of anemia, or history of ischemic disease) [13].

Data were extracted retrospectively from electronic medical records, emergency department documentation, laboratory systems, inpatient charts, and endoscopy reports. Variables included demographics, cirrhosis etiology, baseline laboratory values at presentation (hemoglobin, platelet count, creatinine, bilirubin, albumin, and international normalized ratio—INR), and inpatient course (including ICU admission). Laboratory examinations were performed using Sysmex XN-1000 system (Sysmex Europe, Hamburg, Germany) for hematological determination, respectively Cobas Pure e402 system (Roche Diagnostics, Rotkreuz, Switzerland) for the other biochemical markers.

Liver disease severity was summarized using MELD-Na (Model for End-Stage Liver Disease), ALBI (albumin-to-bilirubin ratio), and PALBI (platelet–albumin–bilirubin) scores, calculated from presentation laboratory values using standard definitions. Established bleeding and mortality risk scores were calculated using published criteria: AIMS65 (albumin, INR, mental status, systolic blood pressure, and age of 65), full Rockall score, and Glasgow Blatchford score [14,15,16]. The full Rockall score incorporated endoscopic diagnosis and stigmata recorded at the index endoscopy (endoscopic component), alongside clinical/physiological variables at presentation.

Based on endoscopic diagnosis fields, bleeding source was categorized as esophageal varices only versus gastric involvement (gastroesophageal varices or isolated gastric varices). Subgroup analyses were prespecified for these categories.

Time to endoscopy was defined as the interval (in hours) from hospital presentation (first recorded time of triage) to the start time of the index endoscopy. This variable was analyzed as a process metric and included in adjusted models.

The primary endpoint was 5-day rebleeding, defined as recurrent bleeding within 120 h of the index endoscopy. Rebleeding required both clinical criteria (new hematemesis and/or melena with hemodynamic deterioration and/or hemoglobin decrease prompting escalation of care and repeat evaluation or transfusion) and endoscopic confirmation of recurrent variceal bleeding or post-banding ulcer bleeding at repeat endoscopy.

Key secondary endpoints were in-hospital mortality (death during the index hospitalization) and 6-week mortality. Six-week mortality was ascertained using a review of hospital and regional medical records supplemented by standardized telephone follow-up performed at approximately 6 weeks after index endoscopy.

Additional endpoints included ICU admission during the index hospitalization and time to endoscopy.

Data collection, storage, and preliminary analysis were performed in Microsoft Excel. Statistical analyses were performed using IBM SPSS Statistics (version 28; IBM Corp., Armonk, NY, USA) and MedCalc Statistical Software (version 22; MedCalc Software Ltd., Ostend, Belgium). Additional analyses and figure generation were performed using Python (version 3.11) with pandas 2.2.0, NumPy 1.26.4, statsmodels 0.14.2, scikit-learn 1.4.2, and Matplotlib 3.5.2.

Prespecified subgroup analyses compared outcomes for esophageal-only varices versus gastric involvement (gastroesophageal or isolated gastric varices). Score discrimination for in-hospital mortality was assessed using ROC curves and AUC with 95% confidence intervals from nonparametric bootstrap resampling (2000 iterations). The effect heterogeneity by variceal location (esophageal-only vs. gastric involvement) was explored using a modality x gastric-involvement interaction term in logistic regression; subgroup estimates were considered hypothesis-generating given limited power. Interaction term results are reported in Section 3 only.

Multivariable logistic regression models evaluated associations between hemostasis modality and each binary endpoint. The primary adjusted model included hemostasis modality (APC vs. EBL), age, MELD-Na, and time to endoscopy. Additional models incorporated ALBI, PALBI, AIMS65, Rockall, and Glasgow Blatchford scores to address liver function and bleeding severity. Adjusted absolute risk differences were estimated via marginal standardization from logistic models.

**Figure A2:**
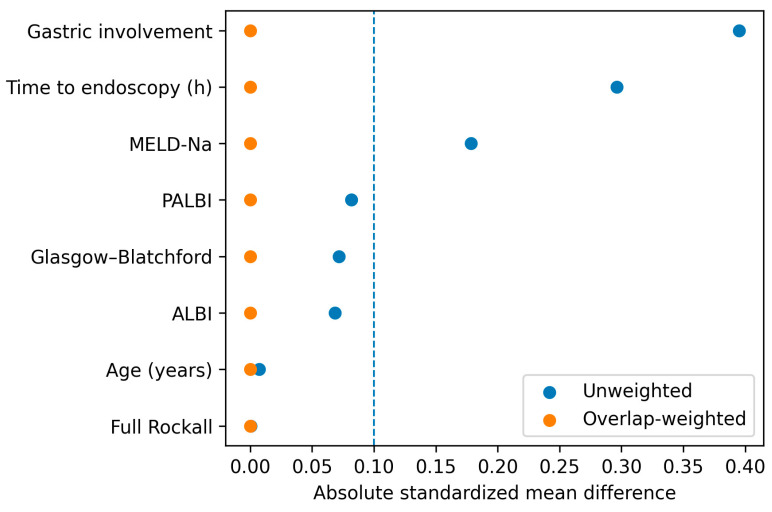
Love plot of absolute standardized mean differences before and after overlap weighting (dashed vertical line at 0.10).

To mitigate confounding by indication in this observational comparison, we performed a prespecified propensity score sensitivity analysis using overlap weighting. The propensity score for receiving APC was estimated using logistic regression, including age, MELD-Na, full Rockall score, Glasgow Blatchford score, time to endoscopy, ALBI, PALBI, and variceal location (gastric involvement vs. esophageal-only). Because modality choice and endoscopic findings were recorded during the same index procedure, endoscopic components used in the full Rockall score were considered contemporaneous with exposure assignment rather than downstream outcomes. Overlap weights were defined as 1-PS for APC and PS for EBL, creating a weighted pseudo-population with improved covariate balance. Covariate balance was assessed using standardized mean differences (SMDs) computed in the overlap-weighted pseudo-population (Appendix A Table A1) and visualized with a Love plot (Appendix A Figure A2). Weighted associations between hemostasis modality and binary outcomes were estimated using weighted logistic regression with robust standard errors. Weighted absolute risk differences were estimated from weighted outcome risks, and 95% confidence intervals were obtained by nonparametric bootstrap resampling (500 iterations), refitting the propensity model within each bootstrap sample. Standardized mean differences were computed on the overlap-weighted pseudo-population as the difference in weighted means divided by the pooled weighted standard deviation, with values reported to four decimals and values < 0.001 displayed as “<0.001” [17,18].

No missing data were present for variables included in prespecified models; therefore, complete-case analyses included all eligible patients. Supportive-care variables (vasoactive therapy with terlipressin) were abstracted from medication administration records and are reported with denominators reflecting documentation.

Continuous variables are presented as mean (standard deviation, SD) and compared using Welch’s *t*-tests. Categorical variables are presented as n (%) and compared using chi-square tests (or Fisher’s exact test when appropriate).

This study was conducted in accordance with the Declaration of Helsinki and institutional standards for research involving human participants. The protocol was reviewed by the Mureș County Emergency Clinical Hospital’s Medical Ethics Commission for Clinical Studies, approval number Ad 34800/4 February 2026, and was granted approval. Because this was a retrospective analysis of routinely collected clinical data with minimal risk to participants, the requirement for informed consent was waived by the reviewing board. All data were de-identified prior to analysis and handled in accordance with applicable data protection policies.

## 3. Results

The baseline characteristics of the study cohort and risk scores are shown in Table 1. Time to endoscopy was similar between groups.

**Figure 1:**
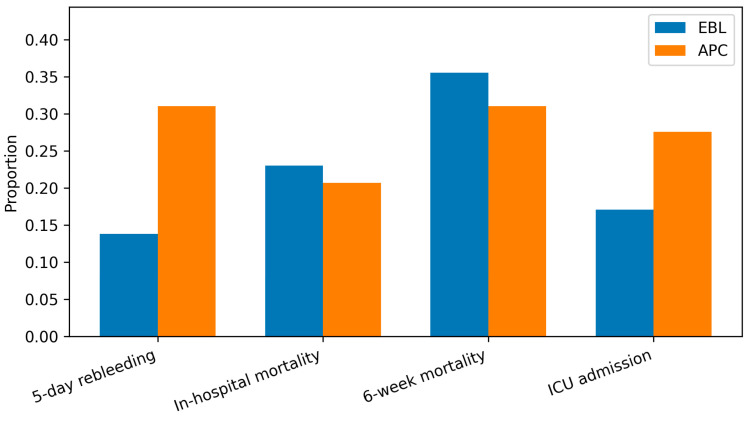
Clinical outcomes by hemostasis modality (unadjusted proportions). (*n* = 181; EBL *n* = 152, APC *n* = 29, proportions reported in Table 2). Bars represent proportions; denominators are group totals.

For the primary endpoint, 5-day rebleeding occurred more frequently after APC than EBL (Table 2, Figure 1). In the primary adjusted model (age, MELD-Na, time to endoscopy), APC was associated with higher odds of 5-day rebleeding. Unadjusted group comparisons (Fisher’s exact) yielded *p* = 0.030 for 5-day rebleeding, *p* = 1.000 for in-hospital mortality, *p* = 0.832 for 6-week mortality, and *p* = 0.199 for ICU admission.

In-hospital and 6-week mortality were similar between groups. ICU admission was numerically higher after APC but not significantly different after adjustment. We evaluated outcomes stratified by esophageal-only vs. gastric involvement (Table 3). Associations were directionally similar, though precision was limited by small APC subgroup sizes.

Exploratory interaction testing for the primary endpoint suggested possible effect heterogeneity by variceal location (modality and gastric involvement *p* for interaction = 0.055), but estimates were imprecise due to small APC counts and should be considered hypothesis-generating. Given the exploratory nature of subgroup and interaction analyses and the small APC subgroup, we did not adjust *p*-values for multiplicity; interaction findings should be interpreted cautiously.

**Figure 2:**
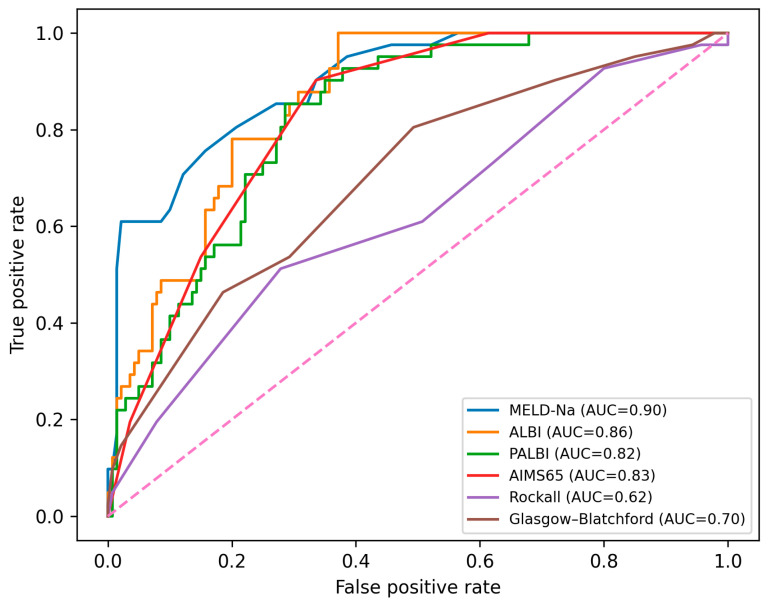
Discrimination for in-hospital mortality for candidate scores (*n* = 181; in-hospital deaths *n* = 41). ROC curves plot sensitivity vs. 1-specificity; AUCs and 95% CIs are reported in Table 4.

The ROC/AUC discrimination for in-hospital mortality is summarized in Table 4 and illustrated in Figure 2. MELD-Na and ALBI provided the highest discrimination in this cohort.

Propensity overlap-weighted sensitivity analysis yielded qualitatively similar findings. In the overlap-weighted pseudo-population, APC was associated with higher 5-day rebleeding (weighted OR 2.17, 95% CI 0.53–8.84), corresponding to an absolute rebleeding risk difference of +13.6 percentage points (95% CI −4.6 to +30.7). Weighted in-hospital mortality risks were 20.9% for APC and 28.5% for EBL (risk difference −7.6 percentage points; 95% CI −18.7 to +4.0), and weighted 6-week mortality risks were 31.1% vs. 39.9% (risk difference −8.8 percentage points; 95% CI −20.2 to +3.2). Sensitivity analysis results are summarized in Table 5.

**Figure 3:**
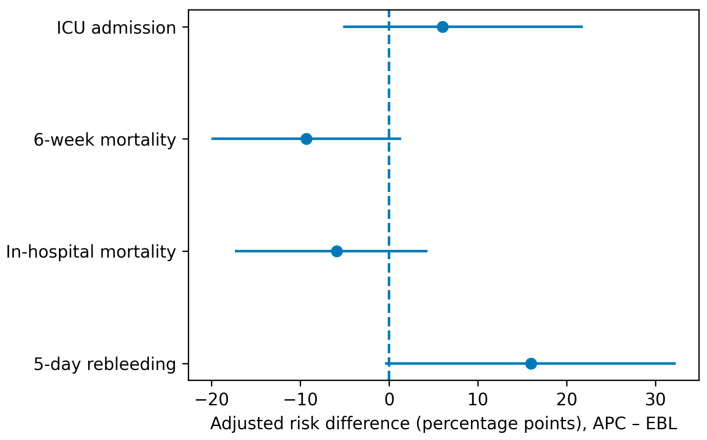
Adjusted risk differences (APC-EBL) for key endpoints estimated by marginal standardization from logistic regression models adjusted for age, MELD-Na, and time to endoscopy (*n* = 181, percentage points reported in Table 6). Error bars indicate 95% confidence intervals. The vertical dashed line denotes no difference (0 percentage points).

After adjustment for age, MELD-Na, and time to endoscopy, the APC strategy remained associated with a higher probability of early recurrent bleeding compared with EBL. Adjusted analyses are summarized in Table 6 and Figure 3. In marginally standardized estimates, the adjusted risk difference (APC-EBL) for 5-day rebleeding was positive, indicating excess early rebleeding in the APC group (Table 6, Figure 3). In contrast, adjusted differences in in-hospital mortality and 6-week mortality were small and not directionally consistent with a large mortality penalty attributable to APC in this observational cohort (Table 6, Figure 3). Likewise, the adjusted risk difference for ICU admission suggested no clinically meaningful increase in ICU utilization associated with APC after accounting for baseline liver disease severity and endoscopy timing.

Figure 3 visualizes the adjusted risk differences and corresponding 95% confidence intervals, highlighting that the most prominent adjusted separation between strategies occurred for early rebleeding, whereas confidence intervals for mortality outcomes overlapped the null. Collectively, these adjusted findings support the interpretation that APC was preferentially applied in higher-complexity bleeding scenarios (e.g., suboptimal conditions for band deployment), with early hemostasis durability potentially lower than EBL, yet without an observable increase in short-term mortality once key severity covariates and time to endoscopy were incorporated (Table 6, Figure 3).

## 4. Discussion

In this retrospective cohort of adults with cirrhosis and endoscopically confirmed AVB, APC used as a rescue or alternative strategy when EBL was not feasible or was judged unsafe was associated with a higher risk of early (5-day) rebleeding, while in-hospital and 6-week mortality were similar to standard EBL after adjustment for age, MELD-Na, and time to endoscopy. These findings are clinically plausible given that APC was preferentially selected in technically challenging scenarios (poor visualization during massive bleeding) and in bleeding patterns where ligation is often suboptimal (gastric varices and post-banding ulcer bleeding) [1,2,3,19].

Current consensus documents emphasize a management bundle for AVB, including vasoactive therapy, antibiotic prophylaxis, restrictive transfusion strategy, and early endoscopy with endoscopic therapy tailored to variceal type [1,2,3,20,21]. EBL remains first-line for esophageal variceal bleeding, whereas gastric variceal hemorrhage generally requires tissue adhesive injection or other specialized techniques. Banding may be less effective and can be technically difficult in active gastric bleeding [2,9,10]. Within this framework, APC should not be considered a replacement for standard-of-care therapies but rather an endoscopic adjunct or rescue modality when immediate mechanical therapy is not achievable. Beyond acute hemostasis, recent reviews highlight broader advances in cirrhosis care, including non-invasive diagnostics and biomarker/AI integration, as well as evolving strategies for complication management (endoscopic therapies, TIPS, transplantation, and regenerative approaches), which may influence future AVB pathways and patient selection for escalation therapies [22].

Compared with published benchmarks for standard EBL-based acute esophageal variceal bleeding care, our unadjusted 5-day rebleeding rate after EBL (13.8%) falls within the ~10–20% early recurrent bleeding range reported in major guideline syntheses, whereas the APC group showed a higher early rebleeding rate (31%). Similarly, our 6-week mortality (35.5% EBL, 31.0% APC) is higher than the 10–20% range commonly cited in contemporary series and guidance, likely reflecting the high proportion of alcohol-related cirrhosis, advanced liver dysfunction (mean MELD-Na ~20), and the tertiary referral care case-mix in our cohort [2,10].

The medical literature on APC in portal hypertension largely evaluates APC as an adjunct to variceal eradication or as a rescue modality rather than as primary therapy for active variceal spurting. In a prospective randomized trial, Cipolletta et al. reported that APC applied to the distal esophageal mucosa after EVL eradication reduced subsequent variceal recurrence compared with observation, supporting the concept that superficial thermal coagulation can promote mucosal fibrosis after mechanical eradication [6]. More recently, Kamal et al. randomized patients after EVL eradication to APC versus observation and observed markedly lower long-term variceal recurrence (21% vs. 68.4%) and less need for rebanding (0% vs. 63.2%) in the APC arm during 30 months of endoscopic follow-up [23]. Smaller consolidation studies (e.g., Furukawa et al.) reported low recurrence in the treated “critical area” without major complications, although sample sizes were limited [24], and a randomized primary-prophylaxis study evaluating a single APC session after EVL found similar short-term recurrence versus EVL alone, suggesting that the APC effect may depend on treatment intensity and protocol design [25]. In contrast, evidence for APC as an acute hemostatic option in AVB is sparse and mostly limited to rescue scenarios, such as EVL-induced ulcer bleeding or diffuse oozing when band deployment is unsafe. In a cohort of EVL-induced ulcer bleeding, APC was used as rescue therapy in a small fraction of cases (3%) alongside repeat EVL, cyanoacrylate sclerotherapy, or balloon tamponade, underscoring that APC is typically a contingency tool rather than definitive variceal therapy [26,27].

The study has several limitations. First, the retrospective single-center design is subject to selection bias and to measurement and ascertainment limitations inherent to routinely collected clinical data. Second, confounding by indication is expected because APC was used selectively when EBL was not feasible or was judged unsafe; residual confounding may persist despite multivariable adjustment and overlap-weighted propensity score sensitivity analysis. Unmeasured factors such as active bleeding severity (spurting/oozing), quality of endoscopic visualization, operator preference, and the relative contribution of post-banding ulcer bleeding could influence both modality choice and early rebleeding. For the observed association with 5-day rebleeding (aOR 2.73), the corresponding E-value is 4.90 (lower 95% CI E-value 1.31), meaning an unmeasured confounder would need a strong association (risk ratio ~4.90 with both APC use and rebleeding) to fully explain away the point estimate. The APC group was small, limiting statistical precision and model stability, particularly for subgroup and interaction analyses. Rebleeding required clinical criteria and endoscopic confirmation, but repeat endoscopy timing and frequency were not systematically captured. Differential thresholds for repeat endoscopy could bias confirmed rebleeding in either direction, and a lower threshold for re-endoscopy in more complex APC cases could increase detection of recurrent bleeding. Finally, the analysis reflects local practice patterns and may not generalize to centers with routine cyanoacrylate injection or EUS-guided therapies for gastric varices; these approaches were not available at our center during the study period. Prospective multicenter studies and causal-inference designs are warranted to better define the role of APC as rescue therapy within guideline-based AVB pathways. Given the imbalanced group sizes (EBL *n* = 152, APC *n* = 29), the study was powered primarily to detect large differences in early rebleeding. Post hoc, with α = 0.05, there is ~80% power to detect an absolute increase in rebleeding from ~14% to ~38% (Δ ≈ 24 percentage points), whereas smaller differences, particularly for mortality, may have been missed. This E-value analysis is presented to quantify robustness to unmeasured confounding, but it does not eliminate the possibility that selective APC use in more complex bleeding (confounding by indication) biased the observed association with early rebleeding.

Strengths of this study include endoscopically confirmed AVB with prespecified endpoints, assessment of multiple validated liver and bleeding severity scores, and a propensity score overlap-weighted sensitivity analysis with formal balance diagnostics.

We incorporated time to endoscopy (hours from presentation) as a process metric in adjusted models. In this cohort, time to endoscopy was not independently associated with mortality after accounting for MELD-Na. This aligns with the concept that liver dysfunction and portal-hypertension severity are dominant drivers of short-term survival, although timely endoscopy remains integral to guideline-based care and facilitates definitive hemostasis [1,2,3].

The MELD-Na score showed the highest discrimination for in-hospital mortality, with ALBI, PALBI, and AIMS65 also demonstrating good performance. Traditional non-variceal upper gastrointestinal bleeding scores (Rockall and Glasgow Blatchford) showed lower discrimination, consistent with prior work indicating that general UGIB scores may perform less well in variceal bleeding populations where hepatic reserve and portal hypertension dominate outcomes [27,28,29,30,31].

For patients in whom EBL is technically difficult or judged unsafe, APC may provide a pragmatic bridge to stabilize bleeding, allowing time for optimization and definitive therapy (repeat endoscopy, cyanoacrylate-based approaches for gastric varices, self-expanding metal stents for refractory esophageal bleeding, or rescue/early TIPS in appropriate high-risk patients) [3,32,33,34]. Given the higher early rebleeding observed, APC-treated patients should be considered higher-surveillance candidates with a low threshold for escalation.

## 5. Conclusions

In adults with cirrhosis and endoscopically confirmed AVB, APC used as a rescue/alternative hemostasis strategy had similar in-hospital and 6-week mortality compared with EBL after adjustment for liver disease severity but was associated with higher 5-day rebleeding. APC may be considered when EBL is technically difficult or judged unsafe, particularly in gastric variceal bleeding or post-banding ulcer bleeding, with careful post-procedure surveillance and readiness to escalate therapy. Importantly, these findings arise from a non-randomized observational comparison and should not be interpreted as establishing a causal treatment effect of APC versus EBL.

In the broader literature, APC has primarily been evaluated as an adjunct after EVL to reduce variceal recurrence and as a rescue option for post-banding ulcer bleeding. Our data extend these observations by quantifying early rebleeding and short-term outcomes when APC is selected as the primary recorded hemostasis modality in real-world, high-complexity AVB scenarios. Accordingly, APC should be viewed as a bridge to definitive, guideline-concordant therapy (repeat endoscopy with EVL for esophageal varices, cyanoacrylate/EUS-guided therapy or radiologic shunt/obliteration approaches for gastric varices, early or rescue TIPS in appropriate high-risk patients) rather than as a substitute for standard treatments [2,3,9,10,21].

## Figures and Tables

**Table 1:** Baseline characteristics and severity scores.

Characteristic	EBL (*n* = 152)	APC (*n* = 29)
Age, years	58.92 (11.40)	58.84 (10.97)
Male sex	117 (77.0%)	23 (79.3%)
Etiology: Alcohol-related	127 (83.6%)	24 (82.8%)
Etiology: Viral hepatitis	5 (3.3%)	5 (17.2%)
Etiology: MASLD	9 (5.9%)	0 (0.0%)
Etiology: Autoimmune	8 (5.3%)	0 (0.0%)
Etiology: Vascular	3 (2.0%)	0 (0.0%)
Etiology: Other	0 (0.0%)	0 (0.0%)
Bleeding source: Esophageal-only	130 (85.5%)	20 (69.0%)
Bleeding source: Gastric involvement	22 (14.5%)	9 (31.0%)
MELD-Na	19.51 (8.60)	21.00 (8.24)
ALBI	−1.43 (0.67)	−1.38 (0.70)
PALBI	−1.87 (0.58)	−1.83 (0.43)
AIMS65	2.28 (1.48)	2.38 (1.70)
Rockall	6.41 (1.78)	6.41 (1.68)
Glasgow Blatchford	16.83 (2.04)	16.69 (1.87)
Time to endoscopy, h	6.26 (1.66)	6.86 (2.34)
Terlipressin administered	148/152 (97.4%)	27/29 (93.1%)
Prophylactic antibiotics	152/152 (100.0%)	29/29 (100.0%)

Note: Supportive-care variables (terlipressin administration, antibiotics, transfusion) were abstracted from medication administration records and are reported with denominators reflecting documentation.

**Table 2:** Clinical outcomes.

Outcome	EBL (*n* = 152)	APC (*n* = 29)
5-day rebleeding	21 (13.8%)	9 (31.0%)
In-hospital mortality	35 (23.0%)	6 (20.7%)
6-week mortality	54 (35.5%)	9 (31.0%)
ICU admission	26 (17.1%)	8 (27.6%)

**Table 3:** Subgroup outcomes by variceal location.

Varix Subgroup	Outcome	EBL	APC
Esophageal-only	5-day rebleeding	14 (10.8%)	7 (35.0%)
Esophageal-only	In-hospital mortality	28 (21.5%)	5 (25.0%)
Esophageal-only	6-week mortality	44 (33.8%)	7 (35.0%)
Esophageal-only	ICU admission	22 (16.9%)	6 (30.0%)
Gastric involvement	5-day rebleeding	7 (31.8%)	2 (22.2%)
Gastric involvement	In-hospital mortality	7 (31.8%)	1 (11.1%)
Gastric involvement	6-week mortality	10 (45.5%)	2 (22.2%)
Gastric involvement	ICU admission	4 (18.2%)	2 (22.2%)

**Table 4:** Discrimination of candidate scores for in-hospital mortality.

Score	AUC	95% CI
MELD-Na	0.898	0.845–0.943
ALBI	0.859	0.802–0.909
PALBI	0.824	0.755–0.882
AIMS65	0.830	0.770–0.884
Rockall	0.624	0.526–0.718
Glasgow Blatchford	0.700	0.604–0.787

**Table 5:** Propensity overlap-weighted sensitivity analysis for APC vs. EBL.

Outcome	APC Weighted Risk	EBL Weighted Risk	Risk Difference (pp)	Weighted OR (95% CI)
5-day rebleeding	30.2%	16.6%	+13.6 (−4.6 to +30.7)	2.17 (0.53–8.84)
In-hospital mortality	20.9%	28.5%	−7.6 (−18.7 to +4.0)	0.66 (0.17–2.55)
6-week mortality	31.1%	39.9%	−8.8 (−20.2 to +3.2)	0.68 (0.20–2.27)
ICU admission	27.6%	19.3%	+8.4 (−2.7 to +21.1)	1.60 (0.41–6.32)

**Table 6:** Adjusted associations of APC vs. EBL (logistic regression; age, MELD-Na, time to endoscopy).

Endpoint	Adjusted OR (95% CI) for APC vs. EBL	*p*-Value	Adjusted Risk Difference
5-day rebleeding	2.73 (1.06–7.03)	0.038	16.0 pp (0.9 pp to 33.6 pp); adj risk 30.0% vs. 14.0%
In-hospital mortality	0.51 (0.13–2.01)	0.336	−5.9 pp (−17.4 pp to 4.5 pp); adj risk 17.8% vs. 23.7%
6-week mortality	0.45 (0.14–1.52)	0.199	−9.3 pp (−19.8 pp to 2.8 pp); adj risk 27.0% vs. 36.3%
ICU admission	1.64 (0.54–4.98)	0.386	6.0 pp (−4.6 pp to 17.4 pp); adj risk 23.8% vs. 17.8%

**Figure A1:**
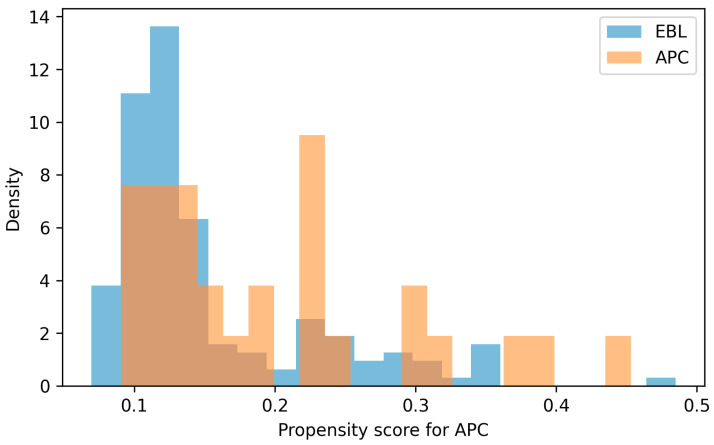
Propensity score overlap for APC treatment assignment by endoscopic modality.

**Figure A3:**
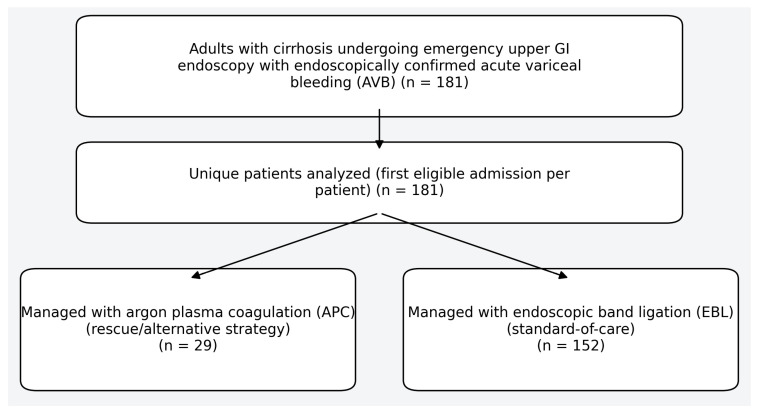
Study flow diagram (STROBE), final analytic sample n = 181, EBL n = 152, APC n = 29).

## Data Availability

De-identified data supporting the findings of this study are available from the corresponding author upon reasonable request, subject to institutional and legal restrictions.

## Abbreviations

The following abbreviations are used in this manuscript:

AIMS65	Albumin, INR, mental status, systolic blood pressure, age ≥ 65 score
ALBI	Albumin–bilirubin score
APC	Argon plasma coagulation
AUC	Area under the curve
AVB	Acute variceal bleeding
CI	Confidence interval
EBL	Endoscopic band ligation
ICU	Intensive care unit
INR	International normalized ratio
MELD-Na	Model for End-Stage Liver Disease—Sodium
OR	Odds ratio
PALBI	Platelet–Albumin–Bilirubin score
PS	Propensity score
ROC	Receiver operating characteristic
SD	Standard deviation
SMD	Standardized mean difference
SPSS	Statistical Package for the Social Sciences
TIPS	Transjugular intrahepatic portosystemic shunt
UGIB	Upper gastrointestinal bleeding

## Appendix A

Covariate balance before and after propensity overlap weighting is shown in Table A1. Means and standardized mean differences are reported to four decimal places, and post-weight SMDs < 0.001 are reported as “<0.001”. Propensity score overlap by exposure group is shown in Figure A1. The Love plot of absolute standardized mean differences before and after overlap weighting is shown in Figure A2.

**Table A1:** Covariate balance.

Covariate	EBL (Unw)	APC (Unw)	SMD (Unw)	EBL (Ow)	APC (Ow)	SMD (Ow)
Age (years)	58.8386	58.9202	−0.0074	58.9832	58.9832	<0.001
MELD-Na	21.0000	19.5132	0.1784	20.7361	20.7361	<0.001
Full Rockall	6.4138	6.4145	−0.0004	6.4293	6.4293	<0.001
Glasgow Blatchford	16.6897	16.8289	−0.0718	16.7055	16.7055	<0.001
Time to endoscopy (h)	6.8552	6.2612	0.2962	6.7146	6.7146	<0.001
ALBI	−1.3809	−1.4275	0.0684	−1.3873	−1.3873	<0.001
PALBI	−1.8700	−1.8300	−0.0818	−1.8500	−1.8500	<0.001
Gastric involvement	0.3103	0.1447	0.3950	0.2679	0.2679	<0.001

A participant flow diagram describing cohort assembly, analytic sample definition, and allocation to endoscopic hemostasis strategy is provided below in Figure A3.
